# Association of spontaneous abortion and *Ureaplasma parvum* detected in placental tissue

**DOI:** 10.1017/S0950268820001302

**Published:** 2020-06-22

**Authors:** C. N. T. Oliveira, M. T. S. Oliveira, H. B. M. Oliveira, L. S. C. Silva, R. S. Freire, M. N. Santos Júnior, M. V. Oliveira, J. Timenetsky, G. B. Campos, L. M. Marques

**Affiliations:** 1 Microbiology and Immunology Laboratory, Federal University of Bahia, Anísio Teixeira Campus, Vitória da Conquista, Bahia, Brazil; 2 Santa Cruz State University, Ilhéus, Bahia, Brazil; 3 Department of Microbiology, Institute of Biomedical Science, University of São Paulo, São Paulo, Brazil

**Keywords:** Miscarriage, *Mollicutes*, reproductive tract infections, ureaplasma infections

## Abstract

Spontaneous abortion is considered a public health problem having several causes, including infections. Among the infectious agents, bacteria of the vaginal microbiota and *Ureaplasma parvum* have been associated with abortion, but their participation needs to be further elucidated. This study aims to evaluate the influence of *Mollicutes* on the development of spontaneous abortion. Women who underwent spontaneous abortion and those with normal birth (control) were studied. Samples of cervical mucus (CM) and placental tissue were collected to identify *Mollicutes* using the quantitative polymerase chain reaction methodology. Eighty-nine women who had a miscarriage and 20 women with normal pregnancies were studied. The presence of *Mollicutes* in placental tissue increased the chance of developing miscarriage sevenfold. The prevalence of *U. parvum* in women who experienced spontaneous abortion was 66.3% in placental tissue. A positive association was observed between the detection of *U. parvum* in samples of placental tissue and abortion. There was a significant increase in microbial load in placental tissue for *M. hominis, U. urealyticum* and *U. parvum* compared to the control group. Detection of *U. parvum* in CM in pregnant women can ascend to the region of the placental tissue and trigger a spontaneous abortion.

## Introduction

Recurrent spontaneous abortion (RSA) or induced abortion (IA) is an important worldwide health outcome, which occurs in about 14% of pregnancy-related deaths due to complications of spontaneous abortion and resulting in about 40 000 pregnancy-related annual deaths. [1]. RSA is defined as two or more successive abortions before 20 weeks from the last menstruation [2]. The possible causes for RSA include parental chromosomal abnormalities, untreated hypothyroidism, uncontrolled diabetes mellitus, certain uterine anatomic abnormalities and antiphospholipid antibody syndrome (APS). Other probable or possible aetiologies include additional endocrine disorders, heritable and/or acquired thrombophilias, immunologic abnormalities, environmental factors and infections. However, the role of infectious agents in recurrent loss is less clear, with a proposed incidence of 0.5–5% [3]. Septic abortion is an infection of the placental tissue and foetus. Placental tissue infection favours it spreading to the uterus, blood and organs. This sepsis causes most maternal deaths in developing countries mainly due to illegal and spontaneous abortions. The fatality rates of septic abortion are extremely variable, ranging from 5% to 20% in many developing countries [1].

Septic abortion is usually caused by bacteria of the vaginal microbiota [1]. Endometrial immunocytes are activated by recurrent bacteria and followed by an intense immune response disturbing the beginning of a pregnancy. This results in excessive Th1 cytokine production. Successful pregnancy is associated with a Th2 cytokine predominance, with Th1 cytokines being detrimental. Therefore, the bacterial toxins or undesirable local cytokines cause uterine contractions, placenta insufficiency, fetal death or life-threatening malformations and amnionitis, which causes abortion in the first trimester as well as preterm labour in the third trimester [4].

Potentially treatable genital tract infections account for 15% of early miscarriages and 66% of late miscarriages [5]. In pregnancy, bacterial vaginosis (BV) is a modification of the vaginal microbiota characterised by a diminished or absent microbiota of lactobacilli, which increases the vaginal pH and a significantly increased colonisation of several anaerobic or facultative microorganisms, mainly *Gardenerella vaginalis*, *Prevotella* sp, *Bacteroides* sp, *Mobiluncus* sp, gram positive cocci and genital mycoplasma (*Mycoplasma hominis* and *Ureaplasma urealyticum*) [6, 7]. Mycoplasmas are prokaryotes with no cell wall and are among the smallest organisms with autonomous growth. They have smaller genomes than most bacteria and require very complex culture media. [8]. They are often present in vulvovaginal microbiota and can be associated with genital disorders [9, 10].

*U. urealyticum* and *U. parvum* are frequently found in the human urogenital tract in both healthy individuals and symptomatic patients [11] and have been isolated in the genital tract from healthy asymptomatic pregnant women and amniotic fluid [12]. *M. genitalium* is associated with male urethritis cervicitis and an increased risk of pelvic inflammatory disease (PID), endometritis and infertility. In the pregnant women, these species have been associated with chorioamnionitis [13]. The chronicity and pathogenesis of this association include evasion of the local host immune response. *M. hominis* can be isolated from endometrial tissue of healthy, nonpregnant women. This may disturb embryonic implantation and therefore, early pregnancy [7, 14].

There is growing evidence of an association between *U. urealyticum*, alone or in combination with *M. hominis* and obstetric complications such as premature rupture of membranes, preterm delivery and abortion. Indeed, maternal inflammatory responses are more intense in intra-amniotic infection with genital mycoplasmas than with other microorganisms [15]. However, there is controversy regarding the specific role of each mycoplasmas species in adverse pregnancy outcomes [9]. Due to the fact that *Mollicutes* infection often presents asymptomatically, being difficult to diagnosis and the literature reports an association of these microorganisms as spontaneous abortion, in addition to the variation in prevalence in different parts of the world and the absence of this in pregnant women in the studied area, the aim of the study was to evaluate the influence of *Mollicutes* on the development of spontaneous abortion.

## Methods

### Population

The cross-sectional case-control study included 89 women who had experienced spontaneous abortion and 20 women with no abortion experience. The clinical samples were obtained from July 2017 to August 2018 in a maternal and child referral centre in Vitória da Conquista Bahia, Brazil. Endocervical swabs before curettage and samples of the removed placental tissue after curettage were analysed. Women over the age of 18 were divided into two groups: with and without spontaneous abortion. Inclusion criteria were pregnancy between 08 and 20 weeks and no previous use of antibiotics for 2 weeks. Abortion due to anatomical abnormalities were also confirmed by ultrasound images and excluded. The control group consisted of women without spontaneous abortion and who had gestation from 38 to 42 weeks with vaginal delivery and no previous use of antibiotics for 2 weeks.

### Clinical and demographic data

Initially, the research team worked with the team of the health clinics to identify possible eligible patients who presented a confirmation of spontaneous abortion by image examination (ultrasound). Subsequently, a questionnaire was administered to patients. Demographic data included age, ethnicity, marital status, religion, residency, education and income and lifestyle, pathological history, menstrual characteristics, sexual history, obstetric pregnancy and current symptoms of the last 3 months.

### Samples

The patients were prepared for the curettage or childbirth by the hospital's health team and the cervical mucus (CM) samples were swabbed from patients with and without spontaneous abortion (*n* = 109). Samples were collected before prophylactic use of antiseptics. After collecting cervical swab samples, all samples were placed in 5 ml transport media and kept at 4 °C, homogenised, aliquoted to 1 ml and stored at −20 °C. In the abortion group, the placental tissue samples were collected by speculum. In the control group, the placental samples were obtained after natural expulsion. Isopropyl alcohol (70% alcohol contents) was used to wipe the entire foetal part. Subsequently, amniotic membranes were ruptured with sterile scalpels and an internal fragment was collected to prevent contamination from the vaginal canal. Placental tissues from both groups were collected in duplicate and immediately refrigerated at −20 °C for quantitative polymerase chain reaction (qPCR) methodologies.

### Real time PCR

Genomic DNA samples of CM and placental tissue were obtained according to the recommendations of Invitrogen Purelink^TM^ Genomic DNA Kits (Invitrogen, Waltham, MA, USA). Real-time PCR assays were performed in a StepOne Plus real-time PCR cycler (Life Technologies) in a 25 ml final volume with the use of TaqMan Real-Time PCR Master Mix (Thermo Fisher Scientific, Waltham, MA, USA). Positive (DNA of *M. genitalium, M. hominis, U. parvum, U. urealyticum* and *N. gonorrhoeae*), negative control and samples were included. The PCR assays included a negative control (without DNA). Positive control and samples were electrophoresed in duplicate. Labelled probes in the TaqMan format were used to amplify the target gene of *M. hominis* [16], *M. genitalium* [17], *Ureaplasma urealyticum* [18], *U. parvum* [18] and *Neisseria gonorrhoeae* [19]. The standardisation of each microbial DNA for absolute quantitation was obtained from the Microbiology Laboratory of the University of São Paulo/Brazil. The DNA was extracted by the boiling method and quantified by spectrophotometry (NanoDrop ND 100). For each assay, a novel standard curve was used and the following quality parameters were adopted: *r*^2^ ⩾ 0.950, reaction efficiency 95%–105% and slope ~3.32. The absolute quantification of microorganisms was done based on the standard curve.

### Statistical analysis

Clinical and demographic data were analysed with IBM SPSS 21^®^ and EpiInfo 7.2.2.16. software. Descriptive analysis of clinical and epidemiological data was performed by frequency comparison and using Pearson's chi-square test considering *P* < 0.05 and a 95% confidence level. To assess the risk factors associated with bacterial infections, the odds ratio (OR) was calculated and all variables with *P* value <0.20 in univariate analysis were included in multivariate analysis using logistic regression. In the final statistical model, only variables with *P* < 0.05 were considered significant. GraphPad Prism software (version 7) was used to analyse the quantification of microorganisms. First, the Kolmogorov–Smirnov test was applied to evaluate the normal distribution of data. Finally, data were analysed by means. Data were considered significant when *P* < 0.05.

## Results

### Demographic data and prevalence of microorganisms

The mean age of the women was 27.3 years (study group: 28 (±7.3) years; control group: 24 (± 7.9) years). Most were married, lived in an urban area, were over 18 years old and had a low income. Both groups were not physically active, nor did they consume alcohol or tobacco. The mean age of menarche was 12.6 years (study group: 13 (± 1.4); control group: 12 (± 1.4). The mean age of onset of sexual activity was 16.5 years (study group: 17 (± 2.8); control group: 15 (±2.5). It should be noted that 38 women did not have prenatal care during pregnancy and 75 women were pregnant for the first time (Table 1).

**Table 1.** Demographic and clinical data of women with spontaneous abortion and the control group, *n* (%)

Variables	Spontaneous abortion		Control	
	*n*	%	*n*	%
Age (y)				
⩽19	15	16.9	7	35
20–29	37	41.6	7	35
⩾30	37	41.6	6	30
Marital status^a^				
With mate	68	78.2	16	80
Without mate	19	21.8	4	20
Provenance				
Countryside	28	21.3	7	35
Urban area	61	76.4	13	35
Ethnicity				
White	27	30.3	4	20
Non-white	62	69.7	16	80
Religion^b^				
Catholic	49	55.1	9	47.4
Non-catholic	26	29.2	5	26.3
None	14	15.7	5	26.3
Schooling^b^^,^^c^				
None	2	2.3	0	0
Up to 7 years of study	29	33	7	36.8
Over 8 years of study	57	64.8	12	63.2
Income^b,c,d^				
Up to minimum wage	58	65.2	15	78.9
Above minimum wage	30	33.7	4	21.1
Physical activity^c^				
Yes	16	18.2	4	20
No	72	81.8	16	80
Alcohol consumption				
Yes	14	15.7	2	10
No	75	84.3	18	90
Smoking				
Yes	4	4.5	1	5
No	85	95.5	19	95
Onset of sexual activity^e^^,^^f^				
<16 years	30	34.9	10	55.6
16 to 17 years old	28	32.6	6	33.3
⩾18 years	28	32.6	2	11.1
Dyspareunia^c^				
Yes	26	29.5	3	15
No	62	70.5	17	85
Prenatal care				
Yes	52	58.4	19	95
No	37	41.6	1	5
Vaginal discharge				
Yes	58	65.2	12	60
No	31	34.8	8	40
Vaginal itching				
Yes	27	30.3	6	30
No	62	69.7	14	70
Pelvic pain				
Yes	33	37.1	6	30
No	56	62.9	14	70
Dysuria				
Yes	16	18	5	25
No	73	82	15	75
Previous pregnancies				
Yes	25	28.1	9	45
No	64	71.9	11	55

a 2 non-respondent in the abortion group.

b 1 non-respondent in the control group.

c 1 non-respondent in the abortion group.

d Minimum wage of R$954.00 per month.

e 3 non-respondent in the abortion group.

f 2 non-respondent in the control group.

The prevalence of each microorganism for the type of sample in both groups and co-infection are presented in Table 2. All targeted DNA of microorganisms were detected at least once in the samples. The overall prevalence of *Mollicutes* in women experiencing spontaneous abortion was 95.5% in CM and 87.6% in the placental tissue with seven times greater chance of developing abortion in the presence of *Mollicutes* (Table 3). The prevalence of *M. genitalium, M. hominis, U. urealyticum, U. parvum* and *N. gonorrhoeae* in placental tissue was 41.6%, 10.1%, 11.2%, 66.3% and 2.2%, respectively. The control group was allowed for detecting *M. genitalium* and *U. parvum* in 55.0%, 25.0% respectively.

**Table 2.** Prevalence and co-infection of microorganisms and spontaneous abortion

Individual prevalence								
Variables	Cervical mucous				Placental tissue			
	Abortion		Control		Abortion		Control	
	*N*	%	*N*	%	*N*	%	*N*	%
*M. hominis*	11	12.4	3	15	9	10.1	0	0
*M. genitalium*	49	55.1	6	30	37	41.6	11	55
*U. parvum*	68	76.4	13	65	59	66.3	5	25
*U. urealyticum*	25	28.1	3	15	10	11.2	0	0
*N. gonorrhoeae*	0	0	1	5	2	2.2	0	0

Co-infection prevalence					
Variable	*n*	%	Variable	*n*	%
*U. parvum* *+* *U. urealyticum*	7	7.9	*M. hominis* *+* *M. genitalium*	6	6.7
*U. parvum* *+* *M. genitalium*	20	22.5	*M. genitalium* *+* *N. gonorrhoeae*	1	1.1
*U. parvum* *+* *M. hominis*	5	5.6	*U. parvum* *+* *M. genitalium* *+* *U. urealyticum*	3	3.4
*U. urealyticum* *+* *M. genitalium*	5	5.6	*U. parvum* *+* *M. genitalium* *+* *M. hominis*	4	4.5

**Table 3.** Univariate analysis of the occurrence or not of abortion and the presence of *Mollicutes*

Variables	Presence of abortion			Presence of *Mollicutes*		
	OR (IC 95%)	*X*^2^	*P*	OR (IC 95%)	*X*^2^	*P*
Age (y)						
⩽19	0.35 (0.10–1.20)	0.90	0.09	2.55 (0.84–7.74)	2.79	0.09
20 to 29	0.86 (0.26–2.79)	0.06	0.80	**2.54 (1.04–6.22)**	**4.30**	0.04*
#x2A7E;30	1.0			1.0		
Marital status						
Without mate	1.12 (0.33–3.74)	0.03	0.86	1.83 (0.66–5.13)	1.36	0.24
With mate	1.0			1.0		
Colour						
Non-white	0.57 (0.17–1.88)	0.86	0.35	0.80 (0.33–1.94)	0.23	0.63
White	1.0			1.0		
Provenance						
Countryside	0.85 (0.31–2.37)	0.09	0.76	1.10 (0.47–2.55)	0.05	0.82
Urban area	1.0			1.0		
Religion						
None	0.51 (0.15–1.78)	1.12	0.29	0.72 (0.25–2.08)	0.36	0.55
Non-catholic	0.96 (0.29–3.15)	0.00	0.94	1.10 (0.44–2.79)	0.04	0.83
Catholic	1.0			1.0		
Schooling						
None	†			0.57 (0.03–9.48)	0.16	0.69
Up to 7 years of study	0.87 (0.31–2.45)	0.07	0.80	1.14 (0.48–2.65)	0.08	0.77
Over 8 years of study	1.0			1.0		
Income						
Up to minimum wage	0.52 (0.15–1.69)	1.23	0.27	0.81 (0.34–1.92)	0.21	0.64
Above minimum wage	1.0			1.0		
Physical activity						
No	1.13 (0.33–3.82)	0.03	0.85	1.58 (0.59–4.23)	0.84	0.36
Yes	1.0			1.0		
Alcohol consumption						
Yes	1.68 (0.35–8.02)	0.43	0.51	2.74 (0.73–10.2)	2.37	0.12
No	1.0			1.0		
Onset of sexual activity						
<16 years	**0.21 (0.04–1.06)**	**4.06**	0.04*	1.20 (0.44–3.25)	0.13	0.71
16–17 years old	0.33 (0.06–1.80)	1.76	0.19	1.21 (0.43–3.40)	0.13	0.71
#x2A7E;18 years	1.0			1.0		
Dyspareunia						
Yes	2.38 (0.64–8.81)	1.76	0.19	1.69 (0.67–4.30)	1.25	0.26
No	1.0			1.0		
Condom use						
No	†			1.62 (0.09–27.2)	0.11	27.2
Sometimes	†			1.33 (0.05–31.1)	0.03	0.85
Less than half the time	†			2.50 (0.13–45.0)	0.40	0.52
More than half of the time	†			1.66 (0.07–37.7)	0.10	0.75
Yes	†			1.0		
Gestational planning						
No	0.57 (0.19–1.72)	0.99	0.32	1.09 (0.48–2.49)	0.05	0.83
Yes	1.0			1.0		
Prenatal care						
No	**13.52 (1.7–105)**	**9.62**	0.00*	1.93 (0.81–4.58)	2.27	0.13
Yes	1.0			1.0		
Vaginal discharge						
Yes	1.25 (0.46–3.37)	0.19	0.66	2.0 (0.88–4.50)	2.84	0.09
No	1.0			1.0		
Vaginal erythema						
Yes	2.28 (0.48–10.7)	1.13	0.29	1.37 (0.48–3.92)	0.35	0.55
No	1.0			1.0		
Dysuria						
Yes	0.66 (0.21–2.07)	0.52	0.47	1.14 (0.41–3.12)	0.06	0.79
No	1.0			1.0		
Blood in sexual intercourse						
Yes	†			0.36 (0.06–2.24)	1.30	0.25
No	†			1.0		
Genital alteration						
Yes	0.63 (0.16–2.60)	0.40	0.53	3.09 (0.64–14.8)	2.14	0.14
No	1.0			1.0		
Previous pregnancies						
1 gestation	0.48 (0.18–1.29)	2.17	0.14	1.51 (0.63–3.61)	0.87	0.35
+1 pregnancy	1.0			1.0		
Presence of *Mollicutes* in the placental tissue						
Yes	**8.13 (2.66–24.8)**	**16.4**	0.00*	†		
No	1.0			†		
*M. hominis* in CM						
Yes	0.80 (0.20–3.17)	0.10	0.75	†		
No	1.0			†		
*U. parvum* in CM						
Yes	1.74 (0.61–4.93)	1.11	0.29	†		
No	1.0			†		
*U. urealyticum* in CM						
Yes	2.21 (0.60–8.22)	1.47	0.23	†		
No	1.0			†		
*U. parvum* in the placental tissue						
Yes	**5.90 (1.9–17.8)**	**11.5**	0.00*	†		
No	1.0			†		

† Not statistically evaluated; CM, cervical mucous; significant value *P* < 0.5.

In the univariate analysis, no prenatal care, early coitus, the prevalence of *Mollicutes* and *U. parvum* in the placental tissue were significantly associated with spontaneous abortion, *P* < 0.05 (Table 3). Detection of *M. hominis* in the placental tissue was associated with the age <19, not being white, previous pregnancies and the presence of *M. hominis* in CM. The presence of *M. genitalium* was significantly associated with *U. urealyticum* in CM. The presence of *U. urealyticum* in placental tissue was associated with *M. genitalium* in CM. Finally, the presence of *U. parvum* was related to age <29 and bleeding during sexual intercourse. A positive association was observed between *U. parvum* detection in placental tissue samples and miscarriage (OR = 5.90). There were no significant differences in lifestyle, pathological history, sexual history, gestational history, menstrual and obstetric characteristics (Table 4).

**Table 4.** Univariate analysis of the occurrence of abortion and presence of *M. hominis, M. genitalium, U. urealyticum* and *U. parvum*

Variables	Presence of *M. hominis*			Presence of *M. genitalium*			Presence of *U. urealyticum*			Presence of *U. parvum*		
	OR (IC 95%)	*X*^2^	*P*	OR (IC 95%)	*X*^2^	*P*	OR (IC 95%)	*X*^2^	*P*	OR (IC 95%)	*X*^2^	*P*
Age (y)												
⩽19	**9.0 (0.85–94.90)**	**4.49**	0.03*	1.26 (0.45–3.54)	0.20	0.66	2.06 (0.40–10.59)	0.77	0.38	**4.22 (1.02–17.47)**	**4.30**	0.04*
20–29	5.62 (0.62–50.72)	2.90	0.08	0.87 (0.37–2.05)	0.10	0.76	0.73 (0.15–3.50)	0.16	0.69	**3.82 (1.39–10.54)**	**7.06**	0.007*
#x2A7E;30	1.0			1.0			1.0			1.0		
Marital status												
Without mate	3.36 (0.80–14.04)	3.00	0.08	2.29 (0.89–5.88)	3.04	0.08	1.63 (0.38–7.04)	0.44	0.51	1.18 (0.40–3.51)	0.09	0.76
With mate	1.0			1.0			1.0			1.0		
Colour												
Non-white	**4.83 (0.91–25.6)**	**3.91**	0.05*	0.78 (0.34–1.80)	0.33	0.56	1.02 (0.24–4.28)	0.00	0.98	0.76 (0.29–2.03)	0.29	0.59
White	1.0			1.0			1.0			1.0		
Provenance												
Countryside	0.59 (0.11–3.05)	0.40	0.52	0.78 (0.34–1.77)	0.34	0.56	2.44 (0.64–9.21)	1.80	0.18	1.41 (0.53–3.73)	0.48	0.49
Urban area	1.0			1.0			1.0			1.0		
Religion												
None	1.19 (0.21–6.69)	0.04	0.83	**3.81 (1.26–11.5)**	**6.03**	0.01*	0.55 (0.06–5.01)	0.29	0.59	1.05 (0.30–3.60)	0.00	0.94
Non-catholic	0.29 (0.03–2.52)	1.41	0.23	1.27 (0.52–3.10)	0.28	0.60	0.93 (0.21–4.09)	0.01	0.93	1.58 (0.56–4.47)	0.74	0.39
Catholic	1.0			1.0			1.0			1.0		
Schooling												
None	†			†			7.14 (0.40–127.56)	2.35	0.13	0.50 (0.03–8.44)	0.24	0.62
Up to 7 years of study	2.12 (0.48–9.18)			2.20 (0.97–4.99)	3.60	0.06	0.53 (0.10–2.73)	0.59	0.44	0.95 (0.37–2.44)	0.01	0.92
Over 8 years of study	1.0			1.0			1.0			1.0		
Income												
Up to minimum wage	†			1.41 (0.61–3.23)	0.66	0.45	0.75 (0.19–2.89)	0.17	0.68	0.95 (0.37–2.41)	0.01	0.91
Above minimum wage	†			1.0			1.0			1.0		
Physical activity												
No	0.75 (0.14–4.02)	0.11	0.74	1.19 (0.44–3.21)	0.12	0.75	1.88 (0.22–16.2)	0.34	0.56	1.20 (0.39–3.70)	0.10	0.75
Yes	1.0			1.0			1.0			1.0		
Alcohol consumption												
Yes	0.64 (0.07–5.60)	0.16	0.69	0.37 (0.11–1.23)	2.76	0.10	1.40 (0.26–7.39)	0.15	0.69	3.57 (0.78–17.15)	2.80	0.09
No	1.0			1.0			1.0			1.0		
Onset of sexual activity												
<16 years	5.40 (0.59–49.47)	2.68	0.10	1.50 (0.58–3.91)	0.69	0.41	0.51 (0.11–2.37)	0.75	0.39	2.38 (0.79–7.15)	2.45	0.12
16–17 years old	1.80 (0.39–8.32)	0.58	0.45	1.18 (0.44–3.21)	0.11	0.74	0.17 (0.02–1.57)	2.99	0.08	2.60 (0.84–8.07)	2.80	0.09
#x2A7E;18 years	1.0			1.0			1.0			1.0		
Dyspareunia												
Yes	3.45 (0.85–14.09)	3.26	0.07	0.84 (0.36–1.99)	0.15	0.70	0.65 (0.13–3.39)	0.26	0.61	1.24 (0.46–3.30)	0.18	0.67
No	1.0			1.0			1.0			1.0		
Condom use												
No	0.11(0.01–2.02)	3.10	0.08	0.70 (0.04–11.7)	0.06	0.80	†			1.68 (0.09–28.52)	0.13	0.72
Sometimes	†			0.40 (0.01–10.0)	0.32	0.57	†			†		
Less than half the time	0.14 (0.01–2.81)	2.12	0.15	1.33 (0.08–23.5)	0.03	0.84	†			3.17 (0.17–58.70)	0.65	0.42
More than half of the time	†			0.60 (0.02–13.6)	0.10	0.75	†			0.50 (0.02–12.90)	0.18	0.67
Yes	1.0			1.0			1.0			1.0		
Gestational planning												
No	4.52 (0.53–38.55)	2.23	0.13	1.47 (0.65–3.31)	0.85	0.36	1.41 (0.34–5.88)	0.22	0.64	0.93 (0.37–2.33)	0.02	0.88
Yes	1.0			1.0			1.0			1.0		
Prenatal Care												
No	0.68 (0.16–2.90)	0.28	0.60	1.23 (0.55–2.71)	0.26	0.61	1.47 (0.39–5.49)	0.33	0.57	1.10 (0.45–2.70)	0.04	0.82
Yes	1.0			1.0			1.0			1.0		
Vaginal discharge												
Yes	4.80 (0.57–40.29)	2.48	0.12	0.54 (0.24–1.19)	2.37	0.12	2.32 (0.46–11.67)	1.09	0.30	2.16 (0.87–5.38)	2.79	0.09
No	1.0			1.0			1.0			1.0		
Vaginal erythema												
Yes	0.46 (0.05–3.96)	0.54	3.96	0.63 (0.23–1.73)	0.81	0.37	3.09 (0.77–12.44)	2.73	0.10	0.75 (0.26–2.19)	0.27	0.60
No	1.0			1.0			1.0			1.0		
Dysuria												
Yes	2.58 (0.57–11.64)	1.60	0.21	0.57 (0.21–1.56)	1.21	0.27	2.18 (0.50–9.53)	1.10	0.29	0.82 (0.27–2.51)	0.13	0.72
Not	1.0			1.0			1.0			1.0		
Blood in sexual intercourse												
Yes	2.34 (0.23–23.59)	0.55	0.45	1.93 (0.31–12.0)	0.51	0.47	†			**0.11 (0.01–1.07)**	**4.97**	0.02*
No	1.0			1.0			†			1.0		
Genital alteration												
Yes	2.98 (0.52–17.19)	1.62	0.20	0.90 (0.27–3.02)	0.03	0.86	**5.21 (1.07–25.52)**	**4.90**	0.03*	4.54 (0.54–38.2)	2.29	0.13
No	1.0			1.0			1.0			1.0		
Previous pregnancies												
1 gestation	**6.42 (1.46–28.17)**	**7.38**	0.006*	1.19 (0.52–2.70)	0.18	0.67	1.84 (0.47–7.17)	0.79	0.37	2.56 (0.85–7.71)	2.92	0.09
+1 pregnancy	1.0			1.0			1.0			1.0		
*M. hominis* in CM												
Yes	**30 (5.73–157.05)**	**27.3**	0.000*	1.83 (0.59–5.70)	1.12	0.29	0.77 (0.09–6.71)	0.05	0.81	1.41 (0.35–5.76)	0.23	0.63
No	1.0			1.0			1.0			1.0		
*M. genitalium* in CM												
Yes	0.98 (0.32–3.0)	0.00	0.97	†			**2.63 (1.0–6.5)**	**4.56**	0.03*	0.46 (0.19–1.13)	2.88	0.09
No	1.0			†			1.0			1.0		
*U. parvum* in CM												
Yes	1.09 (0.21–5.70)	0.01	0.92	0.72 (0.30–1.71)	0.54	0.46	1.27 (0.25–6.49)	0.08	0.78	2.18 (0.80–5.95)	2.38	0.12
No	1.0			1.0			1.0			1.0		
*U. urealyticum* in CM												
Yes	2.25 (0.55–9.17)	1.33	0.25	**2.49 (1.0–6.0)**	**4.25**	0.04*	**7.91 (1.85–33.75)**	**9.80**	0.001*	0.68 (0.26–1.78)	0.62	0.43
No	1.0			1.0			1.0			1.0		
*U. parvum* in the placental tissue												
Yes	1.02 (0.24–4.39)	0.00	0.98	**0.45 (0.20–0.98)**	**4.12**	0.04*	1.21 (0.29–5.06)	0.07	0.79	†		
No	1.0			1.0			1.0			†		

† Not statistically evaluated; CM, cervical mucous; significant value *P* < 0.5.

In the final logistic regression model (Table 5), a positive association was found between spontaneous abortion and the presence of *U. parvum* in the placental tissue and early onset of sexual activity. Another positive association found was the relationship between *M. hominis* and *U. urealyticum* in CM and their respective placental tissue presence. As well as the association of the presence of *M. genitalium* in the placental tissue with *U. parvum* in the placental tissue and *U. urealyticum* in CM. No statistically significant difference was found in the final logistic regression model with the presence of *U. parvum* in the placental tissue.

**Table 5.** Final logistic regression model by groups of selected variables with abortion, *M. hominis, M. genitalium* and *U. urealyticum*

Presence of abortion		
Variable	OR IC (95%)	*P*
Coital		
<16 years	**6.128 (1.15–32.63)**	0.03*
16–17 years old	4.34 (0.75–25.25)	0.10
#x2A7E;18 years	1.0	
Presence of *U. parvum* in the placental tissue		
Yes	**6.46 (2.0–20.7)**	0.002*
No	1.0	
Model Hosmer Lemeshow: *X*^2^ = 2.07, *P* = 0.72		

Presence of *M. hominis* in the placental tissue		
Variable	OR IC (95%)	*P*
Presence of *M. hominis* in CM		
Yes	**62.19 (6.24–619.6)**	0.000*
No	1.0	
Gestational planning		
Yes	0.19 (0.07–5.62)	0.34
No	1.0	
Model Hosmer Lemeshow: *X*^2^ = 0.33, *P* = 0.85		

Presence of *U. urealyticum* in the placental tissue		
Variable	OR IC (95%)	*P*
Provenance		
Countryside	**0.19 (0.39–0.97)**	0.04*
Urban area	1.0	
Presence of *U. urealyticum* in CM		
Yes	**12.53 (2.48–63.2)**	0.002*
No	1.0	
Presence of vaginal erythema		
Yes	3.87 (0.81–18.4)	0.09
No	1.0	
Model Hosmer Lemeshow: *X*^2^ = 2.74, *P* = 0.60		

Presence of *M. genitalium* in the placental tissue		
Variable	OR IC (95%)	*P*
Presence of *U. parvum* in the placental tissue		
Yes	**0.44 (0.19–0.97)**	0.04*
No	1.0	
Presence of *U. urealyticum* in CM		
Yes	**2.54 (1.03–6.25)**	0.04*
No	1.0	
Model Hosmer Lemeshow: *X*^2^ = 0.15, *P* = 0.93		

CM, cervical mucous; significant value *P* < 0.5.

### Quantification of microorganisms

**Fig. 1.**
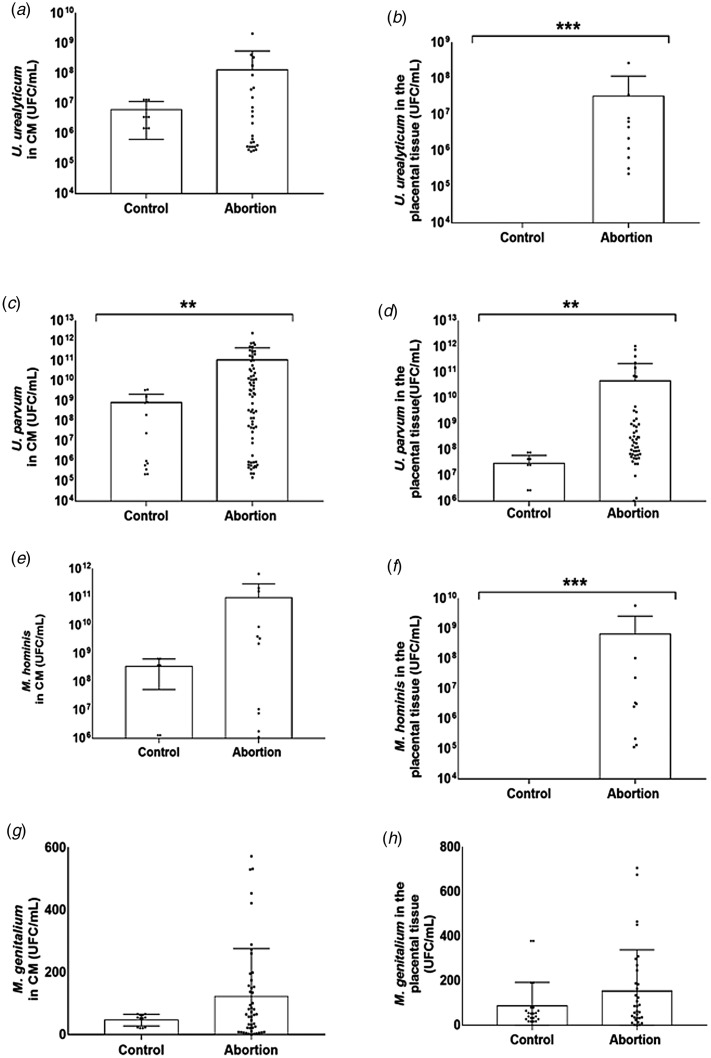
Microbial load of *M. hominis, M. genitalium, U. parvum* and *U. urealyticum* in women with spontaneous abortion and control. (A) *U. urealyticum* in cervical mucous (CFU/ml). (B) *U. urealyticum* in the placental tissue (CFU/mL) *** *P* < 0.0001. (C) *U. parvum* in cervical mucous (CFU/mL) ** *P* < 0.01. (D) *U. parvum* in the placental tissue (CFU/mL) ** *P* < 0.01. (E) *M. hominis* in cervical mucous (CFU/ml). (F) *M. hominis* in the placental tissue (CFU/ml) *** *P* < 0.0001. (G) *M. genitalium* in cervical mucous (CFU/ml); (H) *M. genitalium* in the placental tissue (CFU/ml). Mann–Whitney test. Significant value *P* < 0.5.

A significant increase was observed between *M. hominis* load (mean 6.7 × 10^8^, minimum 1.1 × 10^5^ and maximum 5.9 × 10^9^) *U. parvum* load (mean 4.7 × 10^10^, minimum 1.2 × 10^5^ and maximum 1.0 × 10^12^) and *U. urealyticum* load (mean 3.3 × 10^7^, minimum 2.3 × 10^5^ and maximum 2.7 × 10^8^) detection in placental tissue of women with miscarriage compared to the control group. A significant increase was also observed between *U. parvum* load (mean 1.1 × 10^11^, minimum 1.5 × 10^5^ and maximum 2.5 × 10^12^) detection in CM of women with miscarriage compared to the control group (mean 8.3 × 10^8^, minimum 2.2 × 10^5^ and maximum 3.7 × 10^9^) (Fig. 1).

## Discussion

Spontaneous abortion is a common gestational complication and occurs in approximately 15–20% of pregnancies. This disturbance may also occur due to chromosomal abnormalities, endocrine, immunological changes and infections, which are mostly primarily from the female reproductive tract [20]. Infections in pregnancy cause aggressive inflammatory response mediated mainly by endometrial leukocytes causing spontaneous abortion [4]. The microorganisms associated with amniotic membrane rupture and premature birth are *C. trachomatis, N. gonorrhoeae* and *M. genitalium*. In addition, the presence of *M. hominis, U. urealyticum* and *U. parvum* in the vagina correlates with chorioamnionitis, premature birth and spontaneous abortion; however, a larger and more diverse group of women needs to be studied to better understand spontaneous abortion worldwide [21]. Therefore, it is necessary to understand the prevalence of spontaneous abortion and the risk factors associated with microorganisms, especially mycoplasmas. Although there are many studies in the literature on the association of mycoplasmas with adverse pregnancy events, the results are conflicting often due to the characteristics of the studied population. Therefore, this study investigates the presence of *Mollicutes* in placental tissue and CM in women with and without spontaneous abortion.

In the present study, we identified a protective association (OR = 0.21) in univariate analysis for abortion in women who started sexual activity before age 16. However, in the final logistic regression model, this association did not persist (OR = 6.12) and a higher risk of abortion was identified for women having sex before age 16. Similar results were reported by Machado *et al*., [22] who states that, with the early onset of sex, the time of exposure to sexual activities becomes longer, thereby increasing exposure to various pregnancies. Correia *et al*. [23] also affirm that the beginning of sexual activity in young people is associated with not using contraceptive methods in their first sexual intercourse and, as a consequence, a high number of unplanned pregnancies occur in the first year of sexual activity and spontaneous abortion can occur. A number of these women also underwent unsafe abortion practices possibly due to fear and shame, as well as difficulties in accessing health services or lack of financial resources. A positive association was observed between the occurrence of spontaneous abortion and the absence of prenatal care (OR = 13.5). Machado *et al*. [22] pointed out that not receiving early prenatal care is a risk factor. Not seeking early health services makes it difficult to identify possible treatable causes for spontaneous abortion.

In the present study, we concluded that women with detected *Mollicutes* had a seven times greater chance of developing spontaneous abortion. *U. parvum* was the most prevalent followed by *M. genitalium, M. hominis* and *U. urealyticum*. Other studies on spontaneous abortion mentioned a lower prevalence of *Mollicutes* [20, 24, 25]. In fact, the prevalence of *Mollicutes* in humans varies with age, socioeconomic factors, sexual activity, number of partners and lifestyle [7].

A positive association was observed between *U. parvum* detection in placental tissue samples and miscarriage in univariate analysis (OR = 5.90) and in the final logistic regression model (OR = 6.46). *U. parvum* was associated with age below 19 years (OR = 4.2) and age from 20 to 29 years (OR = 3.8). Studies on abortion [26] and premature delivery [27] also presented comparable data. The ureaplasmal DNA in the placental tissue is related to inflammation, histological changes and elevated cytokine levels, regardless of gestational period [27].

A positive association was also observed in univariate analysis and in the final logistic regression model between the presence of *M. hominis* and *U. urealyticum* with their respective presence in the CM. However, a direct relationship with the development of abortion was not observed. In other studies, the results were also similar [24, 28]. These microorganisms are often found in the genital tract of pregnant women, where they can invade the placenta and contribute to the development of abortion. *U. urealyticum* in asymptomatic pregnant women, in combination with other factors, may be a contributing factor to premature birth and spontaneous abortion [27].

In the present study, there was no association between *M. genitalium* and *N. gonorrhoeae* and spontaneous abortion. Similar results were reported by Ramazanzadeh *et al*. [29] who did not associate *M. genitalium* with spontaneous abortion. These microorganisms cause mucopurulent cervicitis in women and can migrate from the vagina to the uterus. However, an association was observed between the presence of *M. genitalium* in the placental tissue with *U. urealyticum* in CM and *U. parvum* in the placental tissue. In addition, the presence of mycoplasmas with other genital bacteria during preconception and pregnancy may be associated with gestational adverse events including spontaneous abortion [29] and adverse pregnancy outcomes are more severe in patients with more than one organism compared to those with only one [30].

Regarding microbial load, a significant increase was observed in the placental tissue between the amount of *M. hominis* (*P* < 0.00), *U. urealyticum* (*P* < 0.00), *U. parvum* (*P* < 0.01) from the abortion group compared to the control group. This same association was observed in CM only for *U. parvum* (*P* < 0.01). Other studies [27, 31–35] also associated the presence of chorioamnionitis, intense inflammatory reaction and gestational adverse events with the bacterial density of *mollicutes*. However, Contini *et al*. [20] did not associate *M. hominis*, *U. parvum* and *U. urealyticum* density with adverse events. The relationship between *mollicutes* and abortion may be related to their presence in the upper genital tract microbiota and is associated with other risk factors, it may favour massive colonisation, generating aggressive inflammatory response and resulting in spontaneous abortion. [26].

The present study has some limitations. First, the smaller number of participants in the control group was due to numerous refusals of patients in active vaginal labour (transient condition of pain, discomfort and concerns centred on the birth of their children), due to the fact that the collection of information was performed before childbirth. However, in women in the abortion group, no matter how much the situation caused grief, there was an absence of physical pain and a desire to know possible causes associated with abortion. Second, the information about spontaneous abortion was self-reported. And third, in the regression model, there may be a lack of consideration of bacterial vaginosis, due to the fact that *U. parvum*, together with other genital mycoplasmas, is highly associated with BV.

## Conclusion

In this study, we investigated the role of *mollicutes* and co-infection with the spontaneous abortion. This is the first study to show such high placental tissue and CM colonisation rates by *M. hominis, U. urealyticum* and *U. parvum* in women with spontaneous abortion. In addition, *U. parvum* was associated with spontaneous abortion. Moreover, lower genital colonisation in pregnancy by *M. hominis* and *U. urealyticum* was associated with placental tissue infection in women with spontaneous abortion. Thus, it is important to unravel, more broadly, the participation of *mollicutes* in the genesis of abortion.

## Data

The datasets used and/or analysed during the current study are available from the corresponding author on reasonable request.
